# A Prospective Cohort Study Exploring the Joint Influence of Sunlight Exposure and Tanning Bed Use on Basal Cell Carcinoma, Squamous Cell Carcinoma, and Melanoma Risk

**DOI:** 10.21203/rs.3.rs-4005623/v1

**Published:** 2024-03-06

**Authors:** Megan M. Tran, Elisabeth A. George-Washburn, Jongeun Rhee, Wen-Qing Li, Abrar Qureshi, Eunyoung Cho

**Affiliations:** The Warren Alpert Medical School of Brown University; Icahn School of Medicine at Mount Sinai; The Warren Alpert Medical School of Brown University; The Warren Alpert Medical School of Brown University; The Warren Alpert Medical School of Brown University; The Warren Alpert Medical School of Brown University

**Keywords:** Skin cancer risk factors, indoor tanning beds, skin cancer prevention, geospatial analysis (GIS), interaction analysis

## Abstract

Exposure to solar ultraviolet (UV) radiation and use of UV-emitting tanning devices are known risk factors for skin cancer. Few studies have explored the interaction between these risk factors, namely how the risk of skin cancer increases among those who both have been exposed to high levels of natural sunlight and regularly use tanning beds. Nurses’ Health Study II followed 116,430 women, aged 25–42, from 1991 to 2011. Cumulative average UV exposure was based on participants’ residences at follow-up periods. History of severe sunburn during ages 15–20 was used as a proxy for early-life sunlight exposure. Tanning bed use in early life data was collected. Participants reported melanoma, basal cell carcinoma (BCC), and squamous cell carcinoma (SCC) diagnoses. We built multivariable Cox regression models to estimate hazard ratios (HRs) and 95% confidence intervals (CIs) for risk of skin cancer associated with joint effects of sunlight exposure and tanning bed use. Participants with high sunlight exposure and tanning bed use during high school/college had an increased risk of BCC (HR=1.53, CI 1.37–1.71, P interaction =0.01; vs. low UV exposure and no tanning bed use). Participants with a history of severe sunburns and tanning bed use during high school/college were at increased risk of BCC (HR=1.62, CI 1.47–1.79, P interaction =0.02; vs. no sunburns and no tanning bed use). No significant interactions were found between sunlight exposure and tanning bed use on SCC and melanoma risk. We found significant interactions between sunlight exposure and tanning bed use on the risk of BCC.

## Introduction

Skin cancer is the most commonly diagnosed cancer in the United States (US) [27]. Melanoma and nonmelanoma skin cancer incidence rates have been increasing progressively over the past several decades [1, 10]. Ultraviolet (UV) radiation exposure from sunlight and tanning beds are known modifiable risk factors for skin cancer [31]. However, epidemiological studies have not evaluated the risk of skin cancer associated with joint effects of tanning bed use and sunlight exposure. There are also no studies that have investigated whether tanning bed users tend to seek more natural sunlight exposure. As such, skin cancer prevention campaigns may not reflect the true risk profile of engaging in prolonged sunlight exposure combined with tanning bed use.

Individuals who use tanning beds may have misconceptions about the true health risks associated with excess UV exposure that may stem from misinformation. Studies have found that marketing materials from certain tanning companies claim that indoor tanning devices produce a “safer” tan compared to outdoor sunlight [12, 13, 15]. Moreover, some tanning facilities tell customers that exposure to artificial UV radiation from indoor tanning devices “prepares the skin” for outdoor sunlight and prevents burning.

Knowledge about UV radiation from sun exposure, tanning beds and their combined effect on the development of skin cancer are important for skin cancer prevention interventions that aim to reduce skin cancer incidence and decrease associated morbidity and mortality healthcare costs [9]. A study of US high school students showed that three-quarters of those who engaged in indoor tanning had experienced at least one sunburn [17]. Therefore, it is important to measure the interactions between these risk factors on the development of skin cancer to better guide patient education and legislative actions. This study aims to explore the interaction between sunlight exposure and tanning bed use on the risk of melanoma and nonmelanoma skin cancer (NMSC).

## Methods

### Study population

The study population comprised of participants in the Nurses’ Health Study II (NHSII), an ongoing prospective cohort study of female registered nurses [6]. The cohort was established in 1989 with 116,430 registered nurses aged 25 to 42 who responded to a baseline questionnaire on medical conditions and health-related risk factors. This self-administered questionnaire was biennially distributed to participants, with a response rate exceeding 90% at each follow-up cycle [6]. At enrollment, study participants resided in the following 14 states: California, Connecticut, Indiana, Iowa, Kentucky, Massachusetts, Michigan, Missouri, New York, North Carolina, Ohio, Pennsylvania, South Carolina, and Texas. However, during the follow-up period, NHSII participants dynamically moved across the US, resulting in a relatively even distribution of participants across all US states. The study protocol was approved by the institutional review boards of the Brigham and Women’s Hospital and Harvard T.H. Chan School of Public Health, and those of participating registries as required. Completion and return of self-administered questionnaires were considered informed consent.

### Exposure assessment

The 2005 questionnaire cycle collected information on tanning bed use in early life (e.g., none, 1–2 times per year, 3–5 times per year, 6–11 times per year, 12–23 times per year, and ≥ 24 times per year). We focused on the frequency of tanning bed use during high school/college and between ages 25 and 35 years.

Ambient UV exposure was estimated using a spatiotemporal exposure model that applied geostatistical methods to known predictors of UV radiation, such as ozone, aerosol optical depth, cloud cover, and elevation [30]. UV raster cell centroids were intersected with county boundaries and aggregated to the county level using a geographic information system. Using unique US Federal Information Processing Standard codes, county-level data was compiled. Study participants’ mailing addresses for the biennial questionnaire were recorded at baseline and updated at each follow-up period to calculate the cumulative level of average UV exposure during follow-up for each participant. Place of residence for each participant was determined from cycle data from odd-numbered years in the month of June because no data was available mid-cycle. If a participant moved during the follow-up cycle, we assumed that the person spent the entire cycle (2 years) at the residence reported at the end of the cycle. The other measure of sunlight exposure was number of blistering sunburns during young adulthood (ages 15–20), which was included in the baseline questionnaire and used as a proxy for early life sun exposure.

### Outcome measurement

Participants biennially reported new diagnoses of melanoma, basal cell carcinoma (BCC), and squamous cell carcinoma (SCC). Further permission to obtain medical records was requested to confirm cases of melanoma and SCC. The confirmation rates for self-reported melanoma and SCC diagnoses reached 93% and 97%, respectively [6, 32]. Medical records were not obtained for participants who disclosed a diagnosis of BCC, however previous validation studies demonstrated that more than 90% of self-reported BCC cases were confirmed by pathology records [5, 19].

### Covariates

Information on potential risk factors and confounders was collected via the biennial questionnaire. The baseline questionnaire (1989) asked participants to disclose their height and the number of moles on their legs. Information on participant’s weight and smoking status was updated at each follow-up period. Body mass index (BMI) was calculated as weight in kilograms divided by height in meters squared at each follow-up period. The 1991 questionnaire asked about natural hair color at age 20. Family history of melanoma was queried in the baseline questionnaire, and in 1997, 2001, and 2005. Information on physical activity levels was obtained at baseline, and in 1991, 1997, 2001, and 2005; while information on alcohol intake was obtained in 1991, 1995, 1999, 2003, and 2007.

### Inclusion and Exclusion Criteria

NHSII participants who had no baseline cancer history were included in our analysis. Data collected in 1991 was considered baseline for the present study analysis because nearly 13% of participants in 1989 did not report residential address, resulting in missing data on ambient UV exposure. We restricted our analysis to include non-Hispanic White women due to the limited number of non-White participants in the cohort. Participants who died during follow-up, those who did not provide information on residence during follow-up, and those who did not report on tanning bed use during high school/college or between ages 25–35 were excluded from analysis.

## Statistical analysis

Before testing the interaction between sunlight exposure and tanning bed use, the independent effects of cumulative average UV exposure during follow-up, history of blistering sunburns, and tanning bed use on melanoma, BCC, and SCC were examined. Cox proportional hazards models stratified by follow-up cycles were built, adjusting for age (continuous), natural hair color (red, blonde, light brown, dark brown or black), family history of melanoma (yes or no), number of blistering sunburns between ages 15–20 (none, 1–2, 3–4, or ≥ 5), number of moles on legs (none, 1–2, 3–4, or ≥ 5), sunburn reaction during childhood/adolescence (none, some redness, burn, or painful burn/blisters), smoking status (no, past < 10 pack-year, past 10–19 pack-year, past 20 + pack-year, or currently smoking), BMI (< 18.5, 18.5–24.9, 25–29.9, 30–34.9, or ≥ 35 kg/m^2^), and physical activity level (quintiles, metabolic equivalent hours/week). In the models, we accounted for variables that might fluctuate such as ambient UV exposure and smoking status by incorporating data from each two-year questionnaire cycle. Participants were followed from the date of they returned their baseline questionnaire (1991) to the date of first skin cancer diagnosis, death, or the end of the follow-up period (June 2011), whichever came first. Cumulative average UV exposure, blistering sunburn, and tanning bed use were used as continuous variables in trend tests.

To analyze the interaction between tanning bed use and sunlight exposure (cumulative ambient UV exposure and the number of blistering sunburns between ages 15–20), binary exposure variables were created as follows: tanning bed use (yes or no) during high school/college or during ages 25–35, cumulative UV exposure (high or low) using the median value (179 mW/m^2^) as a threshold, and severe sunburn history (yes if ≥ 1 blistering sunburn between ages 15–20 or no if no blistering sunburns between age 15–20). We created interaction terms using the cumulative UV exposure variable (high or low) or the severe sunburn variable (yes or no) with the tanning bed use variable (yes or no) at two time points (high school/college and between ages 25–35). Wald chi-square tests were conducted for the interaction terms using multivariable-adjusted models for each skin cancer type, with the associated p-value interpreted as the p-value for interaction. To examine joint effects, a variable with four indicators was created as follows: 1) low ambient UV exposure (or no severe sunburn history) and no tanning bed use (reference), 2) low ambient UV exposure (or no severe sunburn history) and history of tanning bed use, 3) high ambient UV exposure (or history of severe sunburns) and no tanning bed use, 4) high ambient UV exposure (or history of severe sunburns) and history of tanning bed use. Statistical analyses were performed using SAS software (version 9.4; SAS Institute Inc.). All statistical tests were 2-tailed, the significance level was set at P < 0.05, and 95% confidence intervals (CIs) were calculated.

## Results

Approximately 9.3% of participants (7,077 out of 75,882) used indoor tanning beds at least once during high school/college and 20.0% of participants (15,147 out of 75,885) used indoor tanning beds at least once between ages 25–35 (Table 1). Compared to those who had never used indoor tanning beds, participants who used tanning beds ≥6 times during high school/college were more likely to be younger, to have a family history of melanoma, to have had a blistering sunburn ≥5 times between ages 15–20, to have ≥5 moles on one of their lower extremities, to be a current smoker, and to be physically active. Similar findings were found when evaluating tanning bed use during ages 25–35. In Table 2, we report the independent effects of cumulative average ambient UV exposure, history of blistering sunburns between ages 15–20, and history of tanning bed use during high school/college and during ages 25–35 on the risk of BCC, SCC, and melanoma. Higher cumulative average UV exposure was associated with increased risk of BCC (Hazard Ratio [HR]_Q5_=1.53, 95% CI=1.42, 1.65; P_trend_ <0.0001) and SCC (HR_Q5_=1.61, 95% CI=1.23, 2.11; P_trend_ <0.0001), but was not associated with melanoma risk (P_trend_=0.46). Regarding blistering sunburn history, higher frequency of blistering sunburns between ages 15–20 was associated with increased risk of all skin cancer types (≥5 blistering sunburns vs. none: BCC: HR=1.58, 95% CI=1.45, 1.70; P_trend_ <0.0001, SCC: HR=1.55, 95% CI=1.16, 2.08; P_trend_=0.004, Melanoma: HR=1.76, 95% CI=1.26, 2.40; P_trend_=0.0002). Higher frequency of indoor tanning bed use during high school/college was associated with increased risk of BCC (≥6 uses vs. none: HR=1.70, 95% CI=1.50, 1.92; P_trend_ <0.0001), but was not associated with increased risk of SCC (P_trend_=0.10) or melanoma (P_trend_=0.85). Similarly, more frequent tanning bed use during ages 25–35 was positively associated with both BCC (≥6 uses: HR=1.33, 95% CI=1.22, 1.46; P_trend_ <0.0001) and SCC (≥6 uses: HR=1.62, 95% CI 1.17, 2.24; P_trend_ =0.0004), but was not associated with melanoma (P_trend_=0.28).

Our study also examined the joint effects of sunlight exposure and tanning bed use on skin cancer risk. We identified significant interactions between cumulative average UV exposure during follow-up and tanning bed use during high school/college on the risk of BCC (P_interaction_=0.01) (Table 3). Participants with high ambient UV exposure and a history of tanning bed use had approximately 50% increased risk of BCC compared to participants who had low UV exposure and had never used tanning beds (HR=1.53, 95% CI=1.37, 1.71). While no significant interactions were found between sunlight exposure and tanning bed use on SCC and melanoma risk, the pattern of the associations for SCC was similar to that of BCC.

We also found significant interactions between severe sunburn history and tanning bed use on the risk of BCC (tanning bed use during high school/college: P_interaction_=0.02; during ages 25–35: P_interaction_=0.02) and melanoma (tanning bed use during high school/college: P_interaction_=0.01) as summarized in Table 4. No significant interactions were found between severe sunburn history and tanning bed use during ages 25–25 on the risk of melanoma (P_interaction_=0.18). Women who reported that they had developed a severe sunburn on more than one occasion and used tanning beds during either high school/college (HR=1.62, 95% CI=1.47, 1.79) or during ages 25–35 (HR=1.56, 95% CI=1.43, 1.69) had approximately 60% increased risk of BCC compared to those who had never developed a severe sunburn and never used tanning beds. No significant interactions were found between having had a severe sunburn and tanning bed use on SCC risk. The interaction term was significant for melanoma; however, highest risk of melanoma was associated with those who had no severe sunburns and had used tanning beds (HR=2.33, 95% CI=1.34, 4.06) and those who had experienced a severe sunburn and had never used tanning beds (HR=1.53, 95% CI=1.18, 2.00).

## Discussion

This study examines the interaction between several modifiable skin cancer risk factors including exposure to ambient sunlight, history of severe blistering sunburns between ages 15–20, and use of indoor tanning beds. We identified significant interactions between cumulative ambient sunlight exposure and tanning bed use, as well as severe sunburn history and tanning bed use, on the risk of developing BCC. Specifically, high cumulative levels of UV radiation exposure from sunlight, together with tanning beds use, was associated with 50% increased risk of BCC. Having a history of severe sunburns together with tanning bed use was associated with 60% increased risk of BCC. While no significant interactions were found between sunlight exposure and tanning bed use, as well as between severe sunburn history and tanning bed use, on SCC and melanoma risk, the pattern of the associations were generally similar for SCC. These results suggest that future skin cancer prevention campaigns should focus on discouraging both prolonged outdoor sunlight exposure and indoor tanning bed use. Patients with a history of severe sunburns should be informed that they are at increased risk of skin cancer, and targeted skin cancer prevention interventions may be especially useful for those with multiple behavioral risk factors. Indoor tanning reduction remains an important aspect of skin cancer prevention. While indoor tanning prevalence decreased from 5.5% in 2010 to 3.5% in 2015 [18], the percentage of individuals who reported tanning 25 times or more per year doubled from 12% in 2007 to 24% in 2018 [3, 26].

There is limited evidence of joint effects between outdoor UV exposure and indoor tanning on skin cancer risk. Our study reports that individuals who participated in indoor tanning were more likely to have experienced blistering sunburns between ages 15–20. Some studies have found that people who engage in base tanning via indoor tanning put themselves at higher risk of sunburn [7, 23, 28]. A 2013 study on students at a mid-western university in the US reported that using artificial UV tanning devices during the 10 weeks prior to spring break was associated with an increased risk of sunburn [7]. This association remained significant even after adjusting for sun sensitivity and sunscreen use but became attenuated with additional adjustments for sun exposure measures. Therefore, the study authors concluded that participants who use artificial tanning before vacation may tend to participate in high sun exposure activities.

The biological mechanism linking UV radiation exposure to skin carcinogenesis includes DNA damage such as creation of pyrimidine dimers and oxidative modifications [8, 11, 22]. Mutation formation in specific tumor suppressor genes and oncogenes leads to malignant transformation. At the same time, UV radiation attenuates the ability of host’s immune defense system to recognize and remove malignant cells [2, 4, 29]. Almost all UV-C and much of UV-B emitted by the sun are absorbed by oxygen and ozone in the Earth’s atmosphere. By the time solar UV radiation reaches the Earth’s surface, 95% is UVA and 5% is UVB [20]. Similarly, indoor tanning facilities use devices that emit mostly UVA and some UVB radiation. However, UVA radiation from indoor tanning beds is more intense than that of sunlight, while UVB radiation from indoor tanning beds may approach the intensity of sunlight [14, 25].

While the use of a large population-based cohort with residences in nearly every state across the US lends to the generalizable nature of our findings, this study has several limitations. First, the participants were all registered nurses; as such, they may have more health awareness compared to the general population. Thus, our population’s rates of skin cancer risk behaviors and full body skin examinations may differ from that of the general population. Second, this study only included participants from the NHSII who identified as non-Hispanic White females; thus, the findings from this study may not be generalizable to those of different gender or racial/ethnic groups. The analyses focus on a high-risk population, as women are more likely to sunbathe outdoors and use tanning devices. Moreover, the incidence of skin cancer is highest among non-Hispanic White individuals [16, 21]. Compared to this population, non-White women might experience less access to primary and dermatologic care due to discrimination or other socioeconomic factors [24]. Third, tanning bed use was self-reported and relied on participants accurately recalling their early adulthood behaviors. Therefore, recall bias may impact the quality of our data on tanning bed use frequency, although it is likely to be non-differential. To minimize exposure misclassification, we obtained indoor tanning history from two time points and found that the results were essentially similar in different time periods. Fourth, although melanoma and SCC diagnoses were confirmed, self-reported cases of BCC were not confirmed via medical records. Still, prior studies have shown that more than 90% of self-reported BCC cases were confirmed by pathology records [5, 19].

To our knowledge, this study is the first investigation to quantify the interaction between outdoor UV exposure from sunlight and indoor UV exposure from tanning bed use on skin cancer risk. Our results may serve to combat the myth that “pre-tanning” prior to outdoor sunlight exposure mitigates skin cancer development, which could help inform clinicians, researchers, policy makers, and public health officials in their efforts to prevent skin cancer. This study benefits from the prospective cohort design and large sample size of the NHS II. In addition, this study accounts for potential study participants’ relocations by using updated residential geographic information for each two-year follow-up cycle when estimating cumulative UV exposure. We were similarly able to control for potential time-varying confounders, such as family history of melanoma, physical characteristics, and health behaviors, with the two-year cycle data.

## Conclusion

This study observed that the combinations of high outdoor UV exposure and tanning bed use, as well as severe sunburn history and tanning bed use, increase risk of BCC. These findings can help inform educational and policy-oriented interventions that aim to reduce the incidence, morbidity, mortality, and healthcare costs associated with skin cancer.

## Figures and Tables

**Table 1.** The age-standardized characteristics of skin cancer risk factors by frequency of tanning bed use in the Nurses’ Health Study II in 1991

	Indoor tanning bed use during high school/college (n=75,882)				Indoor tanning bed use at 25–35 years (n=75,885)			
	None (n=68,805; 90.7%)	1–2 (n=3,231; 4.3%)	3–5 (n=1,591; 2.1%)	6+ (n=2,255; 3.0%)	None (n=60,738; 80.0%)	1–2 (n=6,095; 8.0%)	3–5 (n=3,223; 4.2%)	6+ (n=5,829; 7.7%)
Age (years), mean (SD)*	36.5 (4.6)	34.5 (4.9)	34.0 (5.0)	33.3 (4.9)	36.7 (4.6)	34.9 (4.7)	34.4 (4.6)	33.9 (4.6)
Hair color								
Red hair, %	3.9	2.9	4.2	2.8	4.1	3.3	3.1	3.0
Blonde hair, %	16.3	18.1	17.9	19.1	16.2	17.3	18.7	17.8
Light brown hair, %	39.8	40.2	36.5	40.1	39.5	40.5	39.8	41.8
Dark brown hair, %	38.5	37.7	40.6	37.0	38.9	37.6	37.3	36.1
Black hair, %	1.4	1.1	0.7	1.0	1.4	1.3	1.1	1.3
Family history of melanoma, %	4.3	4.8	6.2	5.7	4.4	4.5	3.8	4.4
Number of blistering sunburns between age 15–20 5+, %	9.8	11.2	12.4	15.3	10.0	9.7	10.8	10.5
Number of moles (> 3mm) on lower extremity 5+, %	21.9	24.9	24.8	24.8	21.8	23.2	22.5	23.6
Painful burn or blisters reaction as a child or adolescent, %	24.6	21.8	20.2	20.1	25.5	20.4	18.7	17.3
UV exposure (mW/m^2^), mean (SD)	185.7 (23.7)	186.8 (25.3)	185.3 (24.2)	186.1 (24.8)	186.1 (24.1)	184.5 (22.6)	184.9 (22.8)	184.1 (21.9)
Current smoking, %	11.4	12.8	12.1	14.0	10.6	13.3	13.8	18.4
Body Mass Index (kg/m^2^), mean (SD)	24.6 (5.3)	24.1 (5.1)	24.1 (4.8)	23.9 (4.7)	24.6 (5.4)	23.9 (4.7)	24.1 (4.8)	24.2 (5.0)
Physical activity (metabolic equivalent hours per week), mean (SD)	20.5 (26.1)	21.3 (27.0)	23.0 (28.8)	23.5 (33.7)	20.2 (25.8)	21.7 (26.6)	24.7 (30.9)	23.7 (29.9)

Values are means (standard deviation; SD) for continuous variables, percentages for categorical variables, and are standardized to the age distribution of the study population.

Ultraviolet: UV

* Value is not age adjusted

**Table 2.** Multivariable-adjusted^a^ HRs for basal cell carcinoma (BCC), squamous cell carcinoma (SCC), and melanoma by cumulative average ultraviolet (UV) exposure during follow-up, number of blistering sunburns between ages 15–20, and indoor tanning bed use in the Nurses’ Health Study II (1991–2011)

	BCC		SCC	Melanoma		
Quintile [Q] of UV exposure	Cases, No.	HR (95% CI)	Cases, No.	HR (95% CI)	Cases, No.	HR (95% CI)
Q1 (<168.52 mW/m^2^)	1205	1 (reference)	90	1 (reference)	95	1 (reference)
Q2 (168.52–174.68 mW/m^2^)	1399	1.20 (1.11, 1.29)	84	0.94 (0.70, 1.27)	88	0.96 (0.72, 1.28)
Q3 (174.68–181.78 mW/m^2^)	1461	1.24 (1.15, 1.34)	106	1.18 (0.89, 1.56)	77	0.85 (0.63, 1.15)
Q4 (181.78–206.15 mW/m^2^)	1568	1.37 (1.27, 1.48)	108	1.27 (0.96, 1.68)	89	0.98 (0.73, 1.31)
Q5 (>206.15 mW/m^2^)	1699	1.53 (1.42, 1.65)	138	1.61 (1.23, 2.11)	94	1.04 (0.78, 1.38)
P_trend_		**< 0.0001**		**< 0.0001**		0.46
**Number of blistering sunburns between ages 15–20**						
None	1852	1 (reference)	130	1 (reference)	104	1 (reference)
1–2	2795	1.18 (1.11,1.25)	205	1.22 (0.97,1.52)	169	1.26 (0.98,1.61)
3–4	1570	1.41 (1.31,1.51)	106	1.29 (0.99,1.68)	98	1.55 (1.17,2.07)
≥ 5	1094	1.58 (1.45,1.70)	81	1.55 (1.16,2.08)	69	1.76 (1.26, 2.40)
P_trend_		**< 0.0001**		**0.004**		**0.0002**
**Indoor tanning bed use during high school/college**						
None	5593	1 (reference)	436	1 (reference)	362	1 (reference)
1–2	294	1.19 (1.05, 1.34)	17	0.99 (0.61, 1.60)	24	1.40 (0.92, 2.13)
3–5	135	1.12 (0.94, 1.33)	14	1.72 (1.01, 2.94)	9	1.07 (0.55, 2.08)
≥ 6	273	1.70 (1.50, 1.92)	14	1.32 (0.77, 2.25)	10	0.86 (0.45, 1.61)
P_trend_		**<0.0001**		0.10		0.85
**Indoor tanning bed use during ages 25–35**						
None	4929	1 (reference)	370	1 (reference)	324	1 (reference)
1–2	539	1.17 (1.07, 1.28)	45	1.43 (1.04, 1.95)	29	0.90 (0.61, 1.32)
3–5	299	1.26 (1.12, 1.42)	23	1.49 (0.98, 2.28)	16	0.97 (0.59, 1.61)
≥ 6	539	1.33 (1.22, 1.46)	42	1.62 (1.17, 2.24)	37	1.27 (0.90, 1.80)
P_trend_		**<0.0001**		**0.0004**		0.28

HR: hazard ratio, 95% CI: 95% confidence interval

a Adjusted for age at diagnosis, natural hair color (red, blonde, light brown, dark brown or black), family history of melanoma (yes or no), number of blistering sunburns between age 15 and 20 (none, 1–2, 3–4, or ≥ 5), number of moles on legs (none, 1–2, 3–4, or ≥ 5), sunburn reaction as a child/adolescent (none/some redness, burn, or painful burn/blisters), smoking status (no, past < 10 pack-year, past 10–19 pack-year, past 20+ pack-year, currently smoking), BMI (<18.5, 18.5–24.9, 25–29.9, 30–34.9, and ≥ 35 kg/m^2^), and physical activity (quintiles, metabolic equivalent hours/week).

**Table 3.** Multivariable-adjusted^a^ HRs for basal cell carcinoma (BCC), squamous cell carcinoma (SCC), and melanoma by joint effect between cumulative average ultraviolet (UV) exposure during follow-up^b^ and indoor tanning bed use in the Nurses’ Health Study II (1991–2011)

	BCC		SCC		Melanoma	
Joint effects	Cases, No.	HR (95% CI)	Cases, No.	HR (95% CI)	Cases, No.	HR (95% CI)
**Cumulative average UV and tanning bed use during high school/college**						
Low UV & No tanning bed use	2296	1 (reference)	165	1 (reference)	161	1 (reference)
Low UV & Used tanning bed	314	1.49 (1.32, 1.68)	18	1.37 (0.84, 2.24)	23	1.42 (0.91, 2.21)
High UV & No tanning bed use	3297	1.27 (1.20, 1.34)	271	1.40 (1.15, 1.70)	201	1.10 (0.89, 1.36)
High UV & Used tanning bed	388	1.53 (1.37, 1.71)	27	1.64 (1.08, 2.47)	20	1.06 (0.66, 1.69)
P_interaction_		**0.01**		0.63		0.23
						
**Cumulative average UV and tanning bed use during ages 25–35**						
Low UV & No tanning bed use	2020	1 (reference)	138	1 (reference)	144	1 (reference)
Low UV & Used tanning bed	598	1.27 (1.16, 1.40)	45	1.60 (1.14, 2.24)	41	1.11 (0.78, 1.58)
High UV & No tanning bed use	2909	1.25 (1.18, 1.32)	232	1.41 (1.14, 1.74)	180	1.08 (0.86, 1.34)
High UV & Used tanning bed	779	1.54 (1.42, 1.68)	65	2.07 (1.53, 2.79)	41	1.08 (0.76. 1.54)
P_interaction_		0.65		0.71		0.69

HR: hazard ratio, 95% CI: 95% confidence interval

a Adjusted for age at diagnosis, natural hair color (red, blonde, light brown, dark brown or black), family history of melanoma (yes or no), number of blistering sunburns between age 15 and 20 (none, 1–2, 3–4, or ≥ 5), number of moles on legs (none, 1–2, 3–4, or ≥ 5), sunburn reaction as a child/adolescent (none/some redness, burn, or painful burn/blisters), smoking status (no, past < 10 pack-year, past 10–19 pack-year, past 20+ pack-year, currently smoking), BMI (<18.5, 18.5–24.9, 25–29.9, 30–34.9, and ≥ 35 kg/m^2^), and physical activity (quintiles, metabolic equivalent hours/week).

b The median value (179 mW/m2) was used a cut off for high versus low UV

**Table 4.** Multivariable-adjusted^a^ HRs for basal cell carcinoma (BCC), squamous cell carcinoma (SCC), and melanoma by joint effect between history of severe sunburn^b^ and indoor tanning use in the Nurses’ Health Study II (1991–2011)

	BCC		SCC		Melanoma	
Joint effects	Cases, No.	HR (95% CI)	Cases, No.	HR (95% CI)	Cases, No.	HR (95% CI)
**History of severe sunburn and tanning bed use during high school/college**						
No severe sunburn & No tanning bed use	1471	1 (reference)	111	1 (reference)	81	1 (reference)
No severe sunburn & Used tanning bed	169	1.59 (1.36, 1.87)	8	1.15 (0.56, 2.36)	15	2.33 (1.34, 4.06)
Severe sunburn & No tanning bed use	4113	1.27 (1.20, 1.35)	322	1.29 (1.03, 1.61)	281	1.58 (1.22, 2.03)
Severe sunburn & Used tanning bed	532	1.62 (1.47, 1.79)	37	1.65 (1.13, 2.41)	27	1.40 (0.90, 2.18)
P_interaction_		**0.02**		0.79		**0.01**
						
**History of severe sunburn and tanning bed use during ages 25–35**						
No severe sunburn & No tanning bed use	1282	1 (reference)	88	1 (reference)	74	1 (reference)
No severe sunburn & Used tanning bed	358	1.43 (1.27, 1.61)	31	2.04 (1.35, 3.08)	22	1.41 (0.87, 2.28)
Severe sunburn & No tanning bed use	3640	1.29 (1.21, 1.37)	281	1.41 (1.11, 1.81)	250	1.53 (1.18, 2.00)
Severe sunburn & Used tanning bed	1016	1.56 (1.43, 1.69)	77	1.88 (1.38, 2.57)	59	1.48 (1.04, 2.09)
P_interaction_		**0.02**		0.08		0.18

HR: hazard ratio, 95% CI: 95% confidence interval

a Adjusted for age, natural hair color (red, blonde, light brown, dark brown or black), family history of melanoma (yes or no), number of moles on legs (none, 1–2, 3–4, or ≥ 5), sunburn reaction as a child/adolescent (none/some redness, burn, or painful burn/blisters), smoking status (no, past < 10 pack-year, past 10–19 pack-year, past 20+ pack-year, currently smoking), BMI (<18.5, 18.5–24.9, 25–29.9, 30–34.9, and ≥ 35 kg/m^2^), and physical activity (quintiles, metabolic equivalent hours/week).

b Severe sunburn: ≥ 1 blistering sunburns between age 15–20, no severe sunburn: no bli

## Data Availability

The data of this study are available upon reasonable request. Further information including the procedures to obtain and access data from the Nurses’ Health Studies is described at https://www.nurseshealthstudy.org/researchers (contact e-mail: nhsaccess@channing.harvard.edu).

## Abbreviations

US: United States
UV: Ultraviolet
NMSC: nonmelanoma skin cancer
NHSII: Nurses’ Health Study II
BCC: basal cell carcinoma
SCC: squamous cell carcinoma
BMI: body mass index
HR: Hazard Ratio
CI: Confidence Interval
